# Ionic Liquid Ordering at an Oxide Surface†

**DOI:** 10.1002/cphc.201600774

**Published:** 2016-09-08

**Authors:** Michael Wagstaffe, Mark J. Jackman, Karen L. Syres, Alexander Generalov, Andrew G. Thomas

**Affiliations:** ^1^ School of Materials and Photon Science InstituteThe University of ManchesterOxford RoadManchesterM139PLUK; ^2^ School of Physics and AstronomyThe University of ManchesterOxford RoadManchesterM139PLUK; ^3^ Jeremiah Horrocks InstituteThe University of Central LancashireFylde RoadPrestonPR1 2HEUK; ^4^ MAX IV LaboratoryFotongatan 2225 94LundSweden

**Keywords:** adsorption, ionic liquids, photoelectron spectroscopy, titanium dioxide, X-ray spectroscopy

## Abstract

The interaction of the ionic liquid [C_4_C_1_Im][BF_4_] with anatase TiO_2_, a model photoanode material, has been studied using a combination of synchrotron radiation photoelectron spectroscopy and near‐edge X‐ray absorption fine structure spectroscopy. The system is of interest as a model for fundamental electrolyte–electrode and dye‐sensitized solar cells. The initial interaction involves degradation of the [BF_4_]^−^ anion, resulting in incorporation of F into O vacancies in the anatase surface. At low coverages, [C_4_C_1_Im][BF_4_] is found to order at the anatase(101) surface via electrostatic attraction, with the imidazolium ring oriented 32±4° from the anatase TiO_2_ surface. As the coverage of ionic liquid increases, the influence of the oxide surface on the topmost layers is reduced and the ordering is lost.

Ionic liquids (IL) are room‐temperature (RT) molten salts comprised entirely of cations and anions.^1^ They have an array of unique physico‐chemical properties, including a high ionic conductivity and thermal stability. They are non‐flammable, non‐volatile and their low symmetry leads to low glass transition temperatures. This means they often exist in the liquid phase at room temperature.^2^, ^3^, ^4^ This has led to their use in a wide variety of electrochemical systems, including actuators, corrosion inhibitors, energy storage including batteries and supercapacitors, displays and photovoltaic (PV) devices.^4^, ^5^

Since their introduction in 1991, dye‐sensitized solar cells (DSSCs) have attracted a great deal of interest.^6^, ^7^ DSSCs have mostly used TiO_2_, a wide band gap semiconductor that is non‐toxic, highly resistant to corrosion, has both chemical and mechanical stability and has very low manufacturing costs. The most efficient DSSCs currently use an electrolyte involving a REDOX couple (e.g. I2/I3‐ as the hole transporting medium.^6^ The stability and performance of this electrolyte is critical to the performance of DSSCs and is often a limiting factor in the lifetime of DSSCs. The inherent characteristics of non‐volatile RT ILs make them a highly attractive alternative to more conventional electrolytes, avoiding many of the problems faced when using volatile organic solvents.^3^, ^4^ Experimental solar cells utilizing RT ILs, based on imidazolium cations, as electrolytes have been shown to perform with excellent stability although the short circuit photocurrent was lower than that of typical organic electrolytes.^8^ In addition to their use as electrolytes in photovoltaic devices, ILs have shown great potential as media for the growth of inorganic nanostructures, by anodic oxidation. [C_4_C_1_Im][BF_4_] an imidazolium salt with a BF_4_ counter ion, for example, has been used to grow well defined layers of self‐organized TiO_2_ nanotubes.^3^ It is widely reported that fluorinated molecules lead to the stabilization of reactive [001] facets in anatase TiO_2_ and increase the photocatalytic activity of TiO_2_.^9^, ^10^, ^11^

Both the function and performance of devices employing ILs depend on the behavior of the IL at phase boundaries, interfaces and near interfacial areas.^1^ Interfacial ordering of ILs is thought to reduce the barrier to charge injection in PV devices.^12^, ^13^ An understanding of the interaction between ILs and anatase(101) should help in assessing their suitability for use in PV devices. The (101) surface is the most thermodynamically stable anatase surface and is therefore the dominant surface exposed in nanoparticulate TiO_2_.^14^ Although there is a growing body of work on the surface chemistry of bulk ILs and thin films of ILs on metal surfaces studied by photoelectron spectroscopy,^15^, ^16^, ^17^, ^18^, ^19^, ^20^, ^21^, ^22^, ^23^, ^24^, ^25^, ^26^, ^27^ their interaction with metal oxide solid surfaces is less well studied.^15^, ^16^, ^17^, ^18^, ^28^ In this work we employ a combination of X‐ray photoelectron spectroscopy (XPS) and near‐edge X‐ray absorption fine structure (NEXAFS) spectroscopy to shed light onto the interaction of 1‐butyl‐3‐methylimidazolium tetrafuloroborate ([C_4_C_1_Im][BF_4_]) with the anatase TiO_2_(101) surface.

Figure 2 shows C 1s, N 1s, F 1s and B 1s photoelectron spectra for the 30 Å film of [C_4_C_1_Im][BF_4_] (top, recorded at a substrate temperature of −150 °C) and the 4 Å IL film (bottom, recorded at room temperature). The ionic liquid [C_1_C_4_Im][BF_4_] (≥98 %, Sigma–Aldrich), shown in Figure 1, was deposited on the anatase TiO_2_(101) surface via thermal evaporation in vacuum. The individual elemental spectra are recorded at varying photon energy so that the kinetic energies of the photoemitted electrons are roughly the same. This ensures that an equivalent depth of IL is sampled for all elements. For the full experimental method please see the Supporting Information, S.1. The film thickness was calculated from the attenuation of the Ti 2p signal following adsorption of the ionic liquid, (Figure S.3). The calculation assumes that the IL film grows in a uniform layer‐by‐layer manner.

**Figure 1:**
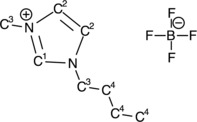
Schematic of [C_4_C_1_Im][BF_4_] highlighting the carbon species that exist in different chemical states.

The XPS core‐level data for the 30 Å film are comparable to those recorded from bulk ILs and therefore this film can be seen to be representative of the bulk IL.^29^ The C 1s spectrum is fitted with four components, with binding energies, 287.8, 287.0, 286.6 and 285.5 eV. These arise from carbon species C1, C2, C3 and C4, respectively, where C1–C4 refer to the labeling in Figure 1. The difference between the chemical environments of C2 and C3 is very small; thus, the resulting chemical shift is only 0.4 eV.^29^, ^30^, ^31^ Therefore, the separation of the peaks was constrained to 0.4 eV and the peak area ratio set to be 1:1. The expected relative intensities of the peaks C1: C2: C3: C4, based on the stoichiometry of the cation, are 0.13:0.25:0.25:0.37. The experimentally observed values of 0.10±0.02:0.24±0.01:0.24±0.01:0.42±0.02 are in good agreement.

There are two nitrogen atoms per ion pair, both located in the ring. The differing length of the side chains does not result in a significant change in the chemical environment so the N 1s spectrum should be fitted with a single species.^29^, ^30^, ^31^ The spectrum is indeed dominated by a component, at a binding energy of 402.3 eV. However, Figure 2 indicates there is some intensity to the lower binding energy side of the main N 1s peak. A further two peaks are required to obtain a satisfactory fit to the data. The origin of these peaks, at binding energies of 400.9 and 399.9 eV is possibly due to a small amount of beam damage. The F 1s and B 1s spectra are each dominated by a single species at binding energies of 686.4 and 194.6 eV, respectively. The measured binding energies and peak assignments in this work are in good agreement with those reported by Foelske‐Schmitz et al.^30^ and Villar‐Garcia et al.,^29^ though we observe a 0.4 eV shift to higher binding energy in all core level spectra, most likely a result of the different methods used to calibrate the binding energy scale (described in S.1). The core‐level spectra and component analysis of the thick film indicate that that [C_4_C_1_Im][BF_4_] can be deposited intact onto the anatase TiO_2_(101) surface, by evaporation at a temperature of around 220 °C, in agreement with a previous adsorption study on Cu.^2^

**Figure 2:**
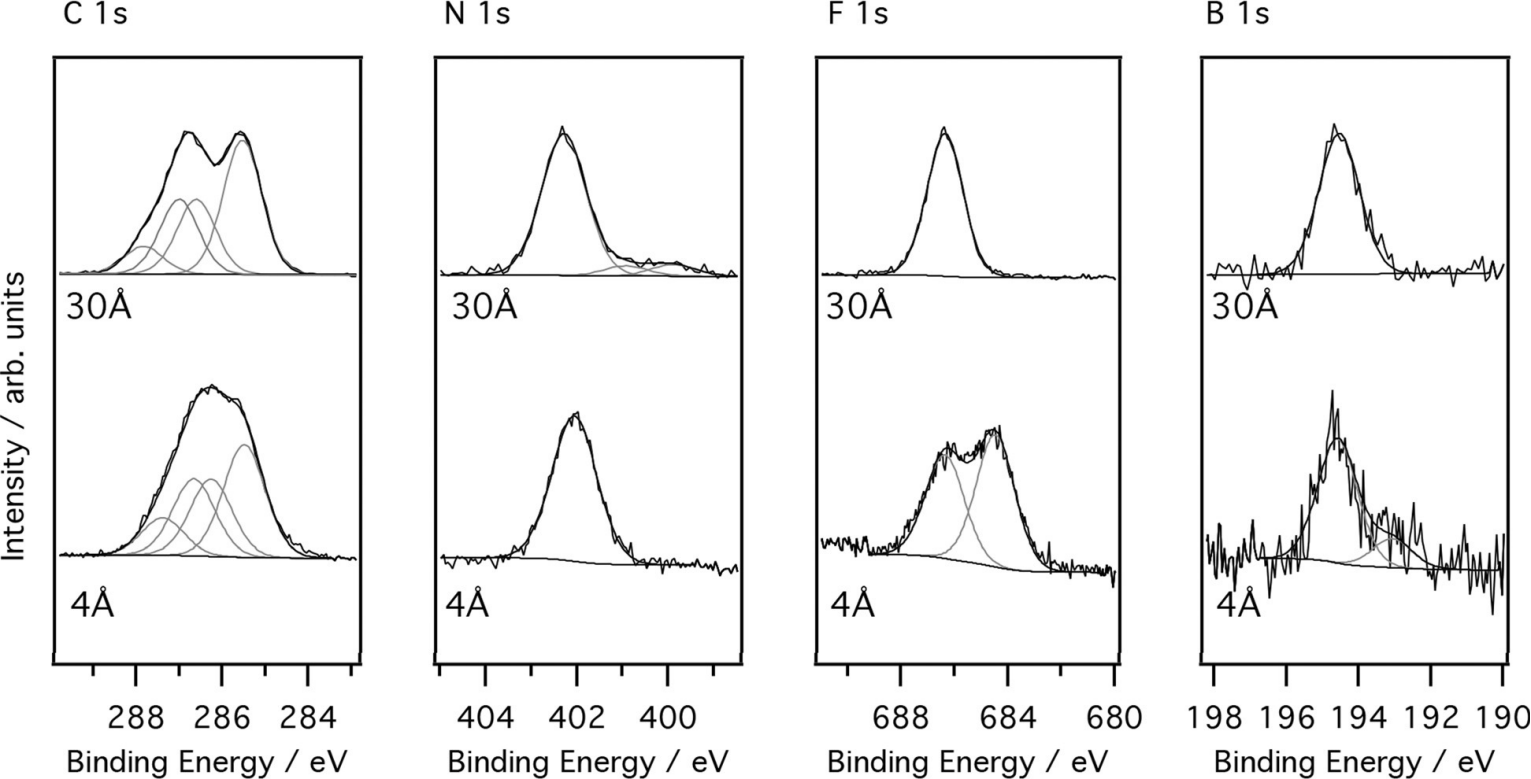
C 1s (*hν*=385 eV), N 1s (*hν*=400 eV), F 1s (*hν*=790 eV) and B 1s (*hν*=300 eV) XPS core‐level spectra showing the anatase TiO_2_(101) single crystal following the adsorption of a 4 Å [C_4_C_1_Im][BF_4_] film (lower curve) and a 30 Å [C_4_C_1_Im][BF_4_] film (upper curve). The spectra are normalized to the maximum peak intensity above the background, this causes an increase in noise for the 4 Å film where there is significantly less IL on the surface. Different photon energies were used to ensure a similar probing depth.

For the 4 Å film, the C 1s spectrum has a different shape, but can still be fitted well with the same four components seen in the thick film. The peak intensity ratios of 0.12±0.02:0.25±0.01:0.25±0.01:0.37±0.02 are also in excellent agreement with the expected stoichiometry. This suggests that the initial [C_4_C_1_Im]^+^ cation layers adsorb intact on the TiO_2_ surface. The components arising from C1, C2 and C3 are shifted by 0.3 eV to lower binding energies relative to the thick film. This binding energy shift is not observed in the peak arising from carbon, C4. The shift to lower binding energy of C1, C2 and C3 derived features can be attributed to the interaction of the imidazolium ring with the surface. It has been reported that the binding energy of molecular orbitals at metal/organic interfaces can vary as a function of the overlayer thickness.^32^ This change in measured binding energy is thought to be a result of electrostatic screening effects on the final state relaxation energy caused by the relative proximity of the adsorbate to the substrate surface. Typically, small shifts to lower binding energy may be expected for all core levels at the interface when compared to the bulk adsorbate.^32^, ^33^ The fact that this shift is not seen in carbon species C4, from the aliphatic chain, suggests that at low coverage the imidazolium ring form an ordered overlayer, lying parallel to the hydrophilic anatase surface, with the aliphatic chain pointed into the vacuum. A shift to higher binding energy of the peak attributed to C4 has been observed by Cremer et al.^27^ who claimed it resulted from the alkyl chain moving from a geometry where it lies parallel to the surface to one in which it is perpendicular to the surface. Such orientations have been seen before, for various ionic liquids on other surfaces, using scanning tunneling microscopy.^16^, ^18^, ^21^, ^22^, ^24^, ^27^ Here we do not observe a shift in binding energy of the C4 peak, suggesting that it does not change conformation in the film thicknesses studies. NEXAFS data discussed below suggest the imidazolium ring lies perpendicular to the surface in the 4 Å film, which is consistent with the lower binding energies of C1, C2 and C3 in the 4 Å film. At 30 Å the IL becomes bulk‐like so that the topmost imidazolium ions are no longer screened by interaction with the surface.

In the F 1s spectrum of the 4 Å film, two peaks are observed at binding energies of 686.4 and 684.6 eV. The lower binding energy peak is consistent with the formation of Ti–F species at the surface.^9^, ^34^, ^35^ A very weak feature is also observed in the B 1s spectrum at a binding energy of 193.1 eV, 1.5 eV lower than the main peak. We believe that upon interaction with the anatase TiO_2_ surface, BF_4_^−^ ions transfer F to the surface, presumably at surface O‐vacancy sites, resulting in the formation of BF_3_. The peak at 193.1 eV in the B 1s spectrum is attributed to BF_3_ trapped in the IL layer.^36^ A peak from BF_3_ is not seen in the F 1s spectra but the reason for this is unclear. It may be due to the fact that the amount of BF_3_ is very low and the peaks from BF_3_ lie beneath the Ti‐F and BF_4_ peaks. The N 1s spectrum shown in Figure 2 shows a similar downward shift in binding energy to that seen in the C 1s spectrum, for carbon species C1, C2 and C3. The peak is located at 402.1 eV, 0.2 eV lower than for the thick film. This observed shift is further evidence of the interaction of the imidazolium ion with the anatase TiO_2_ (101) surface. Other than the degradation of [BF_4_]^−^ resulting in the formation of Ti‐F species, the XPS data suggests that the [C_4_C_1_Im][BF_4_] IL undergoes no chemical interaction with the surface and bonds to the anatase(101) surface solely via electrostatic attraction. See S.4, for a table summarizing all peak assignments, their respective binding energies and their relative abundances

Figure 3 a shows angle‐resolved N K‐edge NEXAFS spectra for the 4 Å [C_4_C_1_Im][BF_4_] film adsorbed on anatase TiO_2_(101), recorded using a multichannel partial yield detector. In all spectra, the first sharp resonance occurs at a photon energy of 401.9 eV. This feature arises from a N 1s→π* transition.^37^ A smaller feature is observed at 403.5 eV, due to to the N 1s→ring σ* excitation. The broad resonances at photon energies above 404 eV are assigned to excitations from N 1s to σ* orbitals. The measured values are in good agreement with those of Ehlert et al.^37^ who combined experimental and quantum chemical calculations in a study of [C_4_C_1_Im][I], for both the C K edge and the N K edge. Figure 3 also shows a plot of peak intensity versus incidence angle and the corresponding fit to the equations of Stöhr, for a surface of twofold or higher symmetry.^38^ Fitting the data gives a tilt angle for the plane of the imidazolium ring of 32±4**°** relative to the surface. Analysis of the C K edge data gives a tilt angle in agreement with the N K edge data (Figure S.5). This result is at odds with calculations for adsorption of [C_2_C_1_Im][[SCN] or [C_2_C_1_Im][B(CN)_4_] on the anatase TiO_2_ surface, where both molecules showed some adsorption with the plane of the cation perpendicular to the surface.^39^ However, in the [C_4_C_1_Im] cation studied here, the aliphatic side chain is longer and perhaps more likely to be oriented away from the hydrophilic TiO_2_ surface. Furthermore, the calculations considered a perfect stoichiometric TiO_2_ surface whereas the photoemission data indicates that F ions become attached to the TiO_2_ surface. This may also be a factor in determining the orientation of the cations. For the thick film, NEXAFS spectra in Figure 3 b show that the ordering observed at low coverage is lost. The resonances, however, occur at the same photon energies as seen in the 4 Å film data suggesting the chemistry of the two films is the same. The loss of ordering in the thick film is perhaps not surprising. The influence of the surface will have less effect on the topmost layers therefore one would perhaps expect this loss of ordering. A combined sum‐frequency generation spectroscopy and X‐ray reflectivity study of the bulk‐air interface does not report any ordering of the imidazolium ion relative to the uppermost surface.^40^ It is also possible that because the thick film could only be formed at low temperature −150 °C the ionic liquid molecules lie as they land and cannot reorient. Previous work has established in the temperature regime around −150 °C, below the glass transition temperature of [C_4_C_1_Im][BF_4_],^41^ the IL adsorbs on the surface via a simultaneous‐multilayer growth mode, in which the neutral ion pairs arrive at the surface in exactly this manner. This results in a frozen IL‐snow structure.^2^

**Figure 3:**
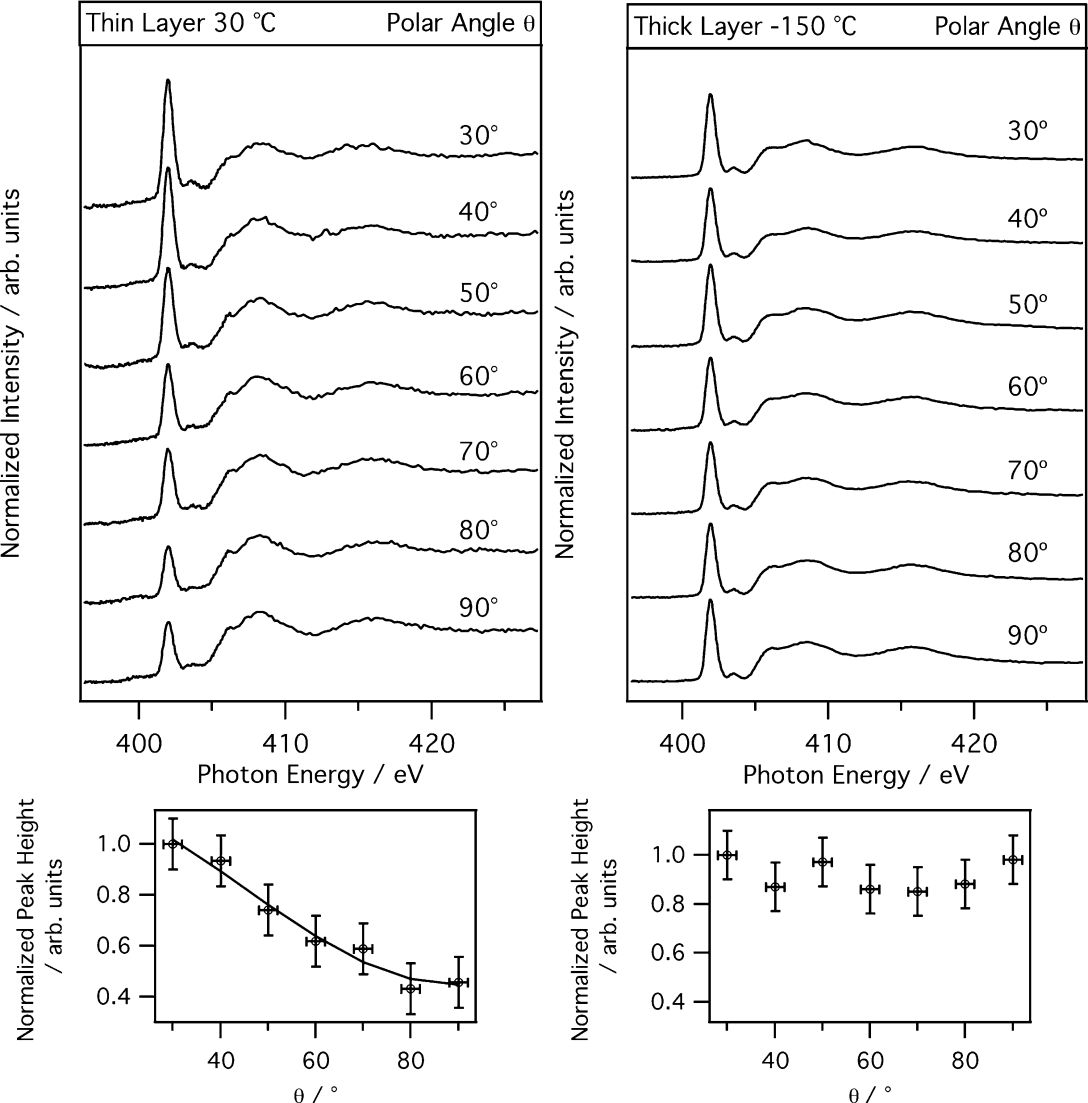
N K‐edge NEXAFS spectra of [C_4_C_1_Im][BF_4_] adsorbed on the anatase TiO_2_(101) surface for: a) the 4 Å IL film and b) the 30 Å IL film. The angle, *θ*, is defined as the angle of the synchrotron beam relative to surface. Included is the corresponding plot of the π* peak intensity as a function of *θ*, the Stöhr equations have been fitted to the data points.^38^

The interaction of [C_4_C_1_Im][BF_4_] with the anatase TiO_2_(101) surface has been investigated using a combination of XPS and NEXAFS spectroscopy. At low coverage, the IL adsorbs in an ordered manner, with the imidizolium ring oriented 32±4 ° from the anatase TiO_2_(101) surface. In addition to this, the [BF_4_]^−^ anion is seen to undergo surface‐induced degradation, leading to the production of Ti‐F species on the surface, most likely reacting with the surface at O‐vacancy sites. This is illustrated schematically in Figure 4. In the 30 Å film ordering is not observed.

**Figure 4:**
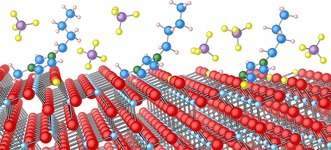
Schematic representation of the interaction of [C_4_C_1_Im][BF_4_] with the anatase TiO_2_(101) surface.

## Supporting information

As a service to our authors and readers, this journal provides supporting information supplied by the authors. Such materials are peer reviewed and may be re‐organized for online delivery, but are not copy‐edited or typeset. Technical support issues arising from supporting information (other than missing files) should be addressed to the authors.

Supplementary
