# Supplementary material for: Ionic Liquid Ordering at an Oxide Surface
Source: Chemphyschem. 2016 Sep 8;17(21):3430–4. doi: 10.1002/cphc.201600774 (PMC5129479; doi:10.1002/cphc.201600774)
Supplement: Supplementary file 1 — Supplementary [file CPHC-17-3430-s001.pdf]

CHEMPHYSCHEM

Supporting Information

Ionic Liquid Ordering at an Oxide Surface**

Michael Wagstaffe,[b] Mark J. Jackman,[b] Karen L. Syres,[c] Alexander Generalov,[d] and Andrew G. Thomas*[a]

cphc_201600774_sm_miscellaneous_information.pdf

S.1 Experimental

The work was carried out on the bending magnet soft X-ray beamline, D1011 (photon energy range 30 eV < hν < 1600 eV) at MAX-lab, Sweden. The anatase $TiO_2(101)$ single crystal (5 mm x 5 mm, Pikem Ltd.), mounted on a Mo sample plate using tantalum wire, was cleaned by repeated 1 keV Ar^+ ion bombardment and 700 °C anneal cycles until a sharp 1 x 1 LEED pattern was obtained and XPS showed the surface to be free of contamination (see supporting information, figure S.2). All photoemission spectra are recorded at normal emission at room temperature for core levels C 1s, N 1s, F 1s, B 1s, Ti 2p and O 1s at photon energies 385 eV, 500 eV, 790 eV, 300 eV, 570 eV and 650 eV respectively. Photoemission spectra are aligned on the binding energy (BE) scale relative to the Ti 2p 3/2 peak at 459.2 eV, corresponding to Ti in the 4+ oxidation state. NEXAFS spectra were recorded, using a multichannel partial yield detector, over both the N K-edge and the C K-edge with incident photon angles, θ, measured relative to the substrate surface plane, ranging between 30° and 90°, in increments of 10°. The partial yield detector was tuned to retard electrons with a kinetic energy of less than 200 eV. Data were recorded with the surface component of the electric vector of the incident radiation at 15 ° from the $[10\bar{1}]$ azimuth with 90%. Horizontal polarization

The ionic liquid $[C_4C_1Im][BF_4]$ (≥98%, Sigma-Aldrich), shown in figure 1, was deposited on the anatase $TiO_2(101)$ surface via thermal evaporation from an evaporator. The ionic liquid was held in a glass crucible in a small vacuum chamber separated from the analysis chamber by a valve. The IL was slowly heated to the evaporation temperature over a period of several hours in order to remove dissolved gases and water. Once the evaporation temperature of 220 °C had been reached, a valve was opened to the chamber and, with the sample facing the evaporator, IL vapor was admitted to the vacuum chamber. The preparation chamber pressure rose to a maximum of 3 x 10^{-7} mbar during evaporation. To obtain a thin layer of ionic liquid on the surface, the substrate was kept at room temperature for 5 minutes. Thicker, multilayer films could be obtained by exposing for longer times but in order to obtain a multilayer film in which photoemission signal from the substrate was completely attenuated, the substrate was cooled to -150 °C using liquid N_2 (LN_2) to reduce the time required to form this film. Measurements from the resulting 30 Å film were also made at -150 °C. Core level spectra were fitted with Gaussian:Lorentzian curves (0.7:0.3) and a Shirley

background. The mean free path length, λ, of photoelectrons in [C_4C_1Im][BF_4] can be determined using the TPP-2M calculation, giving a mean free path length of around 8 Å, at a KE of 110 eV, which corresponds to the KE of Ti 2p photoelectrons in this work.[1]

S.2 Shows the survey scan recorded from the anatase TiO_2(101) surface, at 1000 eV photon energy, following cleaning by repeated sputter and anneal cycles. No surface contamination is observed other than a small amount of residual argon from the sputtering procedure.

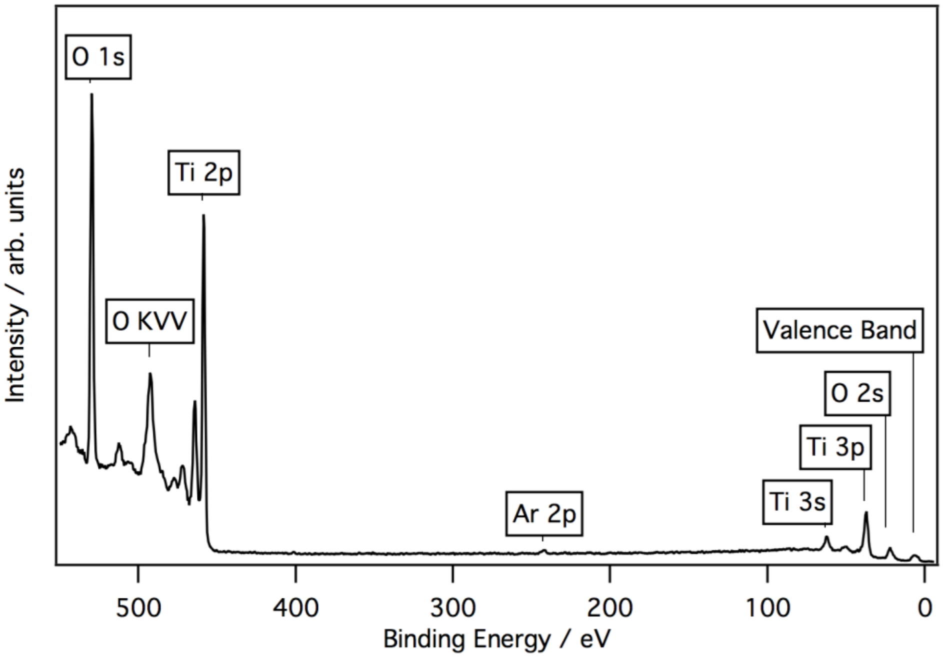

S.3 Ti 2p (photon energy 570 eV) and O 1s (photon energy 650 eV) XPS core-level spectra showing, from bottom to top, the clean anatase TiO_2(101) surface, the anatase TiO_2(101) single crystal following the adsorption of a thin layer of [C_4C_1Im][BF_4] (with the substrate held at room temperature) and a thick layer of [C_4C_1Im][BF_4] (with the substrate held at -150 °C). The spectra are normalized to the maximum peak intensity above the background. The increase in noise levels in the spectra following deposition of the IL is due to attenuation of the signal from the substrate. For the clean surface the Ti 2p specrum is composed of the characteristic spin orbit split peaks at binding energies of 459.2 eV (Ti 2p 3/2) and 465.0 eV (Ti 2p 1/2) as shown in figure 3. These peaks are consistent with Ti4+. A small shoulder

is observed to the lower binding energy side of the main Ti 2p 3/2 peak, due to Ti3+ (457.6 eV), which arises from residual surface oxygen vacancies.[1] The O 1s spectrum for the clean surface is dominated by the large oxide peak at 530.4 eV. A small shoulder is also fitted at a slightly higher binding energy, at 531.6 eV. The origin of this peak has been linked to adsorbed surface hydroxyls and an intrinsic feature in the O 1s spectrum of the anatase TiO_2(101) surface, possibly associated with O-vacancies.[2]

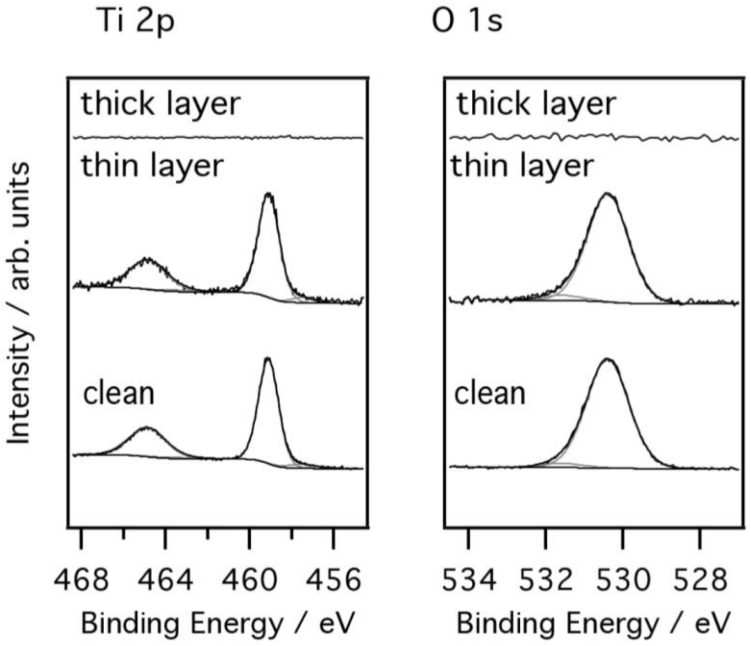

S.4 Peak assignments, their respective binding energies and the relative abundances as a percentage of total peak area that are fitted to the core spectra of elements relating to those present in [C_4C_1Im][BF_4] at low and high coverages. Binding energies are quoted to ±0.1 eV.

Species	Assignment	Low coverage		High coverage	
		BE / eV	%	BE / eV	%
C 1s	C1	287.5	12.3±1. 5	287.8	10.2±1. 6
	C2	286.7	25.4±1. 2	287.0	24.1±1. 1

	C3	286.3	25.4±1.2	286.6	24.1±1.1
	C4	285.5	37.0±1.8	285.5	41.6±1.6
N 1s	Im ring	402.1	100.0	402.3	88.9±2.1
	Beam damage	-	0.0	400.9	5.8±0.9
	Beam damage	-	0.0	399.9	5.3±1.3
F 1s	[B**F**$_4$]$^-$	686.4	44.0±1.8	686.4	100.0
	Ti-**F**	684.6	56.0±1.8	-	0
B 1s	[**B**F$_4$]$^-$	194.6	81.2±4.7	194.6	100.0
	**B**F$_3$	193.1	18.8±4.7	-	0

S.5 Shows the angle resolved C K edge NEXAFS spectra for the thin layer of [C_4C_1Im][BF_4] adsorbed on anatase TiO_2(101) (left). Included is the plot of peak intensity versus incidence angle for a thin layer of [C_4C_1Im][BF_4] (right) and the corresponding fit to the equations of Stöhr for a surface of 2-fold or higher symmetry.[3] The fitting gives a tilt angle for the plane of the imidazolium ring of 34 ± 4° relative to the surface. This angle roughly correlates with the [C_4C_1Im]$^+$ cation lying parallel to the sawtooth structure of the anatase(101) surface and agrees well with NEXAFS spectra from the N K edge.

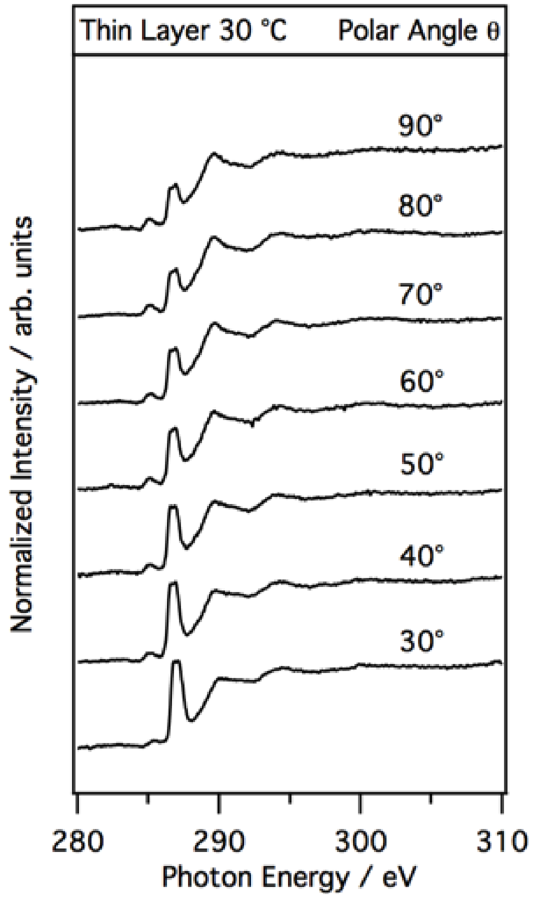

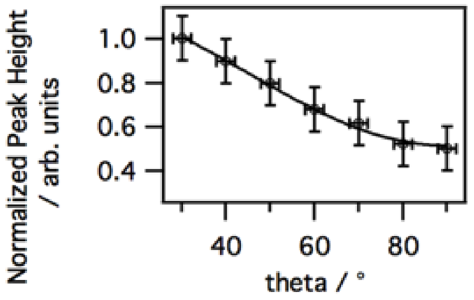

[1] A. Jablonski, *Natl. Inst. Stand. Technol.* **2010**.

[2] M. J. Jackman, A. G. Thomas, C. Muryn, *J. Phys. Chem. C* **2015**, *119*, 13682–13690.

[3] J. Stöhr, *NEXAFS Spectroscopy*, Springer-Verlag: Berlin, **2003**.
